# Supplementary material for: Renin-Angiotensin System in Lung Tumor and Microenvironment Interactions
Source: Cancers (Basel). 2020 Jun 3;12(6):1457. doi: 10.3390/cancers12061457 (PMC7352181; doi:10.3390/cancers12061457)
Supplement: Supplementary file 1 [file cancers-12-01457-s001.pdf]

Supplementary Materials

Renin–Angiotensin System in Lung Tumour and Microenvironment Interactions

Maria Joana Catarata, Ricardo Ribeiro, Maria José Oliveira, Carlos Robalo Cordeiro and Rui Medeiros

Table S1. Clinical Trials of renin angiotensin blockers and its role in lung cancer and pulmonary fibrosis. (adapted from clinicaltrials.gov).

Identifier	Condition	Design	Intervention	Primary Endpoint	Status
**NCT00077064**	Captopril in treating patients with non-small cell lung cancer or limited-stage small cell lung cancer that has been previously treated with radiation therapy with or without chemotherapy	Phase II Randomized Trial	Captopril or placebo	Incidence of pulmonary damage after radiotherapy	Terminated
**NCT01880528**	Lisinopril in reducing shortness of breath caused by radiation therapy in patients with lung cancer	Double-Blind Randomized	Lisinopril or placebo	Incidence of grade 3 or higher hypotension,acute kidney injury, allergic reaction, or anaphylaxis	Completed
**NCT01754909**	Mitigation of radiation pneumonitis and fibrosis	Phase II Randomized Trial	Enalapril or placebo	Occurrence and grade of radiation pneumonitis	Completed
**NCT00879879**	Losartan in treating patients with idiopathic pulmonary fibrosis	Multicenter Clinical Trial	Losartan	Stable or improved forced vital capacity (FVC) response at 1 year in idiophatic pulmonary fibrosis	Completed
**NCT00471562**	Angiotensin-(1–7) in treating patients with metastatic or unresectable solid tumors	Phase I Non-Randomized Clinical Trial	Angiotensin-(1–7) sequentially increasing doses 50–1000 mcg/kg	Maximum tolerated dose and toxicity	Completed
**NCT00886353**	Safety and tolerability study of APN01 (recombinant human angiotensin converting enzyme 2)	Phase II Randomized Trial	APN01 i.v. or placebo	Safety and tolerability profile of APN01	Completed
**NCT00880386**	Losartan in treating pulmonary fibrosis in patients with Stage I, II, or III Non-Small Cell Lung Cancer	Multicenter Clinical Trial	Losartan and radiotherapy	Change in carbon monoxide diffusing capacity (DLCO) at 6 months	Withdrawn

© 2020 by the authors. Licensee MDPI, Basel, Switzerland. This article is an open access article distributed under the terms and conditions of the Creative Commons Attribution (CC BY) license (http://creativecommons.org/licenses/by/4.0/).
